# Exploring Acceptability and Feasibility of Evidence-Based Practice in Child Welfare Settings: A Pilot Study with Attachment-Based Family Therapy

**DOI:** 10.5334/pb.338

**Published:** 2017-04-12

**Authors:** Tara Santens, Suzanne A. Levy, Guy S. Diamond, Caroline Braet, Mildred Vyvey, Elisabeth Heylboeck, Guy Bosmans

**Affiliations:** 1 Parenting and Special Education Research Unit, Catholic University of Leuven (KU Leuven), Leopold Vanderkelenstraat 32, Leuven, BE; 2 Center For Family Intervention Science, College of Nursing and Health Professions, Drexel University, Philadelphia, PA, 19104, US; 3 Department of Developmental, Personality, and Social Psychology, Ghent University, BE; 4 VZW Daidalos (home-based service), BE; 5 VZW aPart team Centrum Ambulante Begeleiding Gent (home-based service), BE

**Keywords:** Child Welfare System, Evidence-Based Practice, Attachment-Based Family Therapy, Implementation, Acceptability, Feasibility

## Abstract

The Flemish Child Welfare System (CWS) is in great need of a shared empirically informed clinical strategy for working with depressed adolescents and their families. Many evidence-based practices (EBP) exist, but little is known as to whether they can be successfully imported in the CWS. Therefore, the current study explores the implementation of a particular EBP, Attachment-Based Family Therapy (ABFT), in home-based services of the Flemish CWS in Belgium. Specifically, the study focused on (1) the acceptability of ABFT by counselors and whether negative attitudes about EBP can be changed (*n* = 73 counselors), and (2) the feasibility of implementing ABFT (*n* = 43 adolescents, 11–17 years old, 72% female) by exploring initial effectiveness. The results suggest that (1) initial negative attitudes of counselors towards ABFT were significantly more positive after attending training and discussions about ABFT, and that (2) ABFT could be used by counselors to successfully reduce adolescent depressive symptoms. Future research should include a control group to draw stronger causal conclusions. Strengths and limitations of the study’s design and implications for further dissemination are discussed.

The Child Welfare System (CWS) is a section in governments that seeks to ensure the health and safety of children. One of the main goals of CWSs is improving family functioning. Unfortunately, to date, there are few well-defined and empirically proven treatment models that have been adopted by CWS services to achieve this goal. One way to respond to this need is to implement evidence-based practices (EBP) that provide a programmatic and empirically supported clinical approach to improve family functioning. EBPs are treatments that integrate the best available research with clinical expertise, taking into account the context, patient characteristics, culture, and preferences for providing services (Beidas & Kendall, 2014). Over time, researchers repeatedly observed the lack of empirically supported, structured, and well-defined treatment models in CWS (Barth et al., 2005, Berry, 1988), which compromises the quality and effectiveness of care (Weisz, Jensen-Doss, & Hawley, 2006; Weisz et al., 2013). Unfortunately, CWS’ incorporation of EBPs to improve family functioning remains limited.

Although strategies and benefits of implementing EBPs in CWS have been suggested (Aarons, Hurlburt, & Horwitz, 2011; Aarons & Palinkas, 2007; Chaffin & Friedrich, 2004; Dawson & Berry, 2002; Kessler, Gira, & Poetner, 2005), little research has investigated whether EBPs can be implemented in the CWS. Therefore, the current study aimed to explore the acceptability and feasibility of a particular EBP in home-based services of the Flemish CWS in Belgium. The treatment that was selected to study was Attachment-Based Family Therapy (ABFT; Diamond, Diamond, & Levy, 2014).

## Home-Based Services of the Flemish Child Welfare System in Belgium

The Flemish^1^ CWS is a large system supporting high-risk children and their families. In 2013, 202 CWS services provided treatment for 27,572 children between 0 and 21 years of age, which comprises 1.48% of the total population of Flemish youth (*n* = 1,857,222). The majority of referred youth are adolescents between 10 and 19 years of age (66.87%) who entered the system because of a problematic home situation (Jongerenwelzijn, 2014). High-risk children preferably first receive help in their home. They are only separated from their families if unsafe circumstances (e.g. abuse, neglect) do not improve. Consequently, the home-based services of the CWS have become important in providing high-risk children and their families treatment through weekly home-visits. Thus, effective home-based interventions aiming to reinstate security and trust in intra-familial relationships would be worth exploring.

Counselors of home-based services typically have to work with the most difficult families that deal with multiple, complex, chronic, socio-economic and/or psychosocial problems. Referrals occur either voluntarily by the CWS referral system or compulsory by Juvenile Court (Grietens, Mercken, Vanderfaeillie, & Loots, 2007). The home-based services’ principal therapeutic mission is to strengthen the resilience of high-risk children and their families by improving problematic family relationships and dynamics. To achieve these goals, they often follow a family systems approach to stimulate communication and parenting skills, and to reinstate security in intra-familial relationships.

Over the past decade, Flemish home-based services have faced several challenges due to the multi-problem profile of referred families. First, multi-problem families are known to be difficult to treat due to constant interpersonal conflicts and crises. This creates an atmosphere in which counselors feel forced to respond to each new urgent crisis without being able to work on underlying (often relational) core problems. In their attempt to provide prompt and appropriate care to these tangible problems and needs, home-based counselors have little opportunity to develop or adopt a uniform and clearly articulated clinical treatment model to help them guide complex daily clinical decision-making. Therefore, home-based services have developed an eclectic treatment approach consisting of a mixture of therapeutic techniques from different theoretical orientations (Stroobants, Vanderfaeillie, & Andries, 2014). Although this “Treatment as Usual” (TAU) approach (i.e. usual clinical care consisting of a broad assortment of interventions that are typically not guided by one particular theoretical orientation and not necessarily supported by empirical evidence) demonstrates the creativity, commitment and investment of these services, there is a great concern about its limited effectiveness (Weiss, Catron, & Harris, 2000; Weiss, Catron, Harris, & Phung, 1999; Weisz et al., 2013; Weisz, Jensen-Doss, & Hawley, 2006). For Flemish home-based services offering short-term treatment trajectories, Stroobants and colleagues (2014) recently confirmed this concern in an effectiveness study showing small effects of usual care on client outcomes (Stroobants et al., 2014). Consequently, it seems reasonable to assume that home-based services of the Flemish CWS could benefit from a more systematic treatment approach that offers a shared framework to increase home-based counselors’ intentionality to get to core issues more quickly and effectively.

The high prevalence of depressed adolescents is a second challenge that complicates home-based services’ daily clinical work (Stroobants et al., 2014). Depression is a serious mental health problem and, can lead to suicide (e.g., Costello, Pine, Hammen, et al., 2002; WHO, 2014). Recent research in Flemish home-based services suggests that for referred families, depending on measure and informant, 22 to 42.5% of the adolescent CWS population has clinically high levels of depressive symptoms. Unfortunately, after home-based guidance these problems remained largely unaltered (Stroobants et al., 2014). Given these small effect sizes and given the need for a more shared family relationship-focused treatment approach, Flemish policy makers decided to implement Attachment-Based Family Therapy (ABFT) as an evidence-based treatment for depressed adolescents and their families (Diamond et al., 2014).

## Attachment-Based Family Therapy

Attachment-Based Family Therapy (ABFT; Diamond, Diamond, & Levy, 2014) is one of the few standardized family therapy models for which empirical evidence supports its efficacy to reduce adolescent depression and suicidal ideation. ABFT is a short-term (16 weeks), task- and principle-driven family psychotherapy model. It builds on the assumption that depressed and suicidal adolescents stopped seeking (emotional) support of their primary caregivers in times of distress due to previous interpersonal disappointments and breaches in trust (Allen & Land, 1999; Bowlby, 1969, 1973, 1980; Cassidy, 2008). Therefore, ABFT aims to repair trust and cooperation between the adolescent and primary caregiver(s), re-establishing the primary caregiver as a source of support for the adolescent to help regulate emotional distress and to promote autonomy.

To achieve these goals, ABFT consists of five treatment tasks (Diamond et al., 2014). First, the Relational Reframe Task (Task 1) sets the foundation for treatment by shifting the family’s focus from “fixing” the adolescent’s symptoms to improving family relationships. The Adolescent Alliance Building Task (Task 2) occurs with the adolescent alone in order to acknowledge and expand his/her narrative and feelings about relational disappointments and unmet attachment needs, and prepare the adolescent to discuss these with his/her parent. The Parent Alliance Building Task (Task 3) occurs with parents alone to empathize with their personal stressors and family-of-origin attachment history that may have affected their parenting style, in order to increase motivation to learn emotion coaching parenting skills to communicate in a new way with the adolescent. These first three tasks set the foundation for the Attachment Task (Task 4) during which in-session, experiential, emotionally arousing, attachment-promoting interactions are engineered. In these conversations the adolescent discloses vulnerable feelings about relational disappointments, and parents respond in a sensitive, supportive, validating, loving and protective manner. Finally, once the foundations of a secure parent-child relationship are (re-)established, the Autonomy Promoting Task (Task 5) focuses on adolescent and parents negotiating autonomy within this revived context of trust of a secure relationship (Diamond et al., 2014).

The efficacy of ABFT has been demonstrated in multiple randomized controlled trials (Diamond, Russon, & Levy, 2016; Diamond et al., 2010; Diamond, Reis, Diamond, Siqueland, & Isaacs, 2002). These studies provide support for ABFT’s success in reducing depression, and have awarded ABFT the designation of an empirically proven program by the Promising Practices Network (2011) and high ratings for the outcomes of depressive symptoms and suicidal ideations as well as readiness for dissemination (3.5–4.0 out of 4.0) in SAMHSA’s National Registry of Evidenced-based Programs and Practices (2013).

To date, little is known about whether counselors in community settings will accept ABFT and whether ABFT is effective in “real-world” or community-based settings. One study in Norway compared ABFT to a TAU group in community-based clinics. Clinic-referred patients were recruited from the intake office, and randomly assigned to ABFT or to TAU. Trained clinicians employed at the local hospitals administered the treatment. The latter study’s design also retained aspects of a typical efficacy study in that it maintained strict inclusion and exclusion criteria and implemented clinical training and supervision of ABFT hospital therapists involved in the study (Israel & Diamond, 2013). Despite this shift from research to clinical settings, participants in the ABFT condition showed a statistically significant reduction in symptoms compared to TAU on the Hamilton Depression Inventory (HAM-D; Hamilton, 1960) with an effect size of 1.08 (Israel & Diamond, 2013). Based on these results, it seemed reasonable to assume that ABFT may be transferable to the context of home-based services of the Flemish CWS as well.

## The Flemish pilot study

The current project started as a result of an invitation of the Flemish government who asked researchers of Ghent University to implement and evaluate EBP in the CWS home-based services. The research team offered the government a list of potentially valuable treatment programs, based on Weisz and colleagues’ overview of evidence-based psychotherapy for children and adolescents with behavioral and/or emotional problems (Weisz & Gray, 2008; Weisz, McCarty, & Valeri, 2006). Out of this list, the Flemish government selected ABFT to implement in CWS because of its’ perceived compatibility and fit within the CWS’s mission and family systems approach to strengthen the resilience of at-risk depressed, suicidal, and/or traumatized children and their families.

The Flemish pilot project started with nationwide meetings with the representatives of the CWS home-based services. During these meetings, CWS counselors expressed skepticism and concerns which overlapped with concerns identified in previous studies about attitudes towards EBPs (e.g. Addis, Wade, & Hatgis, 1999; Kazdin, 2008; Weisz & Gray, 2008; Weisz, Jensen-Doss, & Hawley, 2006). Specifically, counselors worried about (1) using one theoretical model to guide complex daily decision processes, (2) using a brief treatment approach, (3) manualized treatments restricting counselors’ clinical freedom, creativity and personal style of working, and (4) manuals lacking flexibility to deal with individual needs and crises of the complex multi-problem families. In general, counselors doubted that ABFT would fit within their ongoing practices. They expressed suspicion about the government’s possible hidden agenda, fearing that they would be forced to work harder without receiving the necessary economic support for the enhanced workload. In past studies, counselors’ negative attitudes towards EBPs limited counselors’ willingness to implement EBPs (Addis, Wade, & Hatgis, 1999; Kazdin, 2008; Weisz & Gray, 2008; Weisz, Jensen-Doss, & Hawley, 2006). Consequently, in order to enhance implementation success of ABFT in Flemish home-based services of CWS, we aimed to explore whether we could increase counselors’ acceptability of EBP as part of a broader implementation plan. To account for ceiling effects, and given that especially the group of most critical counselors can have a negative impact on successful implementation, we explored whether attending the workshop not only influenced attitudes overall in favor of EBP, but specifically benefited attitudes of those counselors who initially had the most negative attitudes (Research goal 1).

This implementation plan consisted of three phases. For phase one, we organized a free one-day introductory workshop open to all home-based service counselors and administrators. Two main strategies aimed to overcome their concerns. First, we took to heart Pagoto and colleagues’ (2007) suggestion that misconceptions and misunderstanding about EBPs require teaching counselors about the content and goals of EBPs. To address this, we organized a lecture by the ABFT developers during which the ABFT model was introduced. Second, we considered Kendall and Beidas’ (2007) suggestion that the gap between science and practice might be overcome if counselors can share their concerns regarding manual-based treatments. Therefore, we organized a discussion between the ABFT developers and the CWS counselors and administrators. These strategies aimed to improve counselors’ understanding of the manuals, and give them an active voice in the implementation process (Addis & Krasnow, 2000). To explore the impact of these strategies, we measured counselors’ and administrators’ attitudes towards EBPs in general and ABFT specifically before and after the one-day introductory workshop (Research Question 1).

In phase two of the implementation plan, we organized two additional workshop days to provide a more in-depth training for agencies that expressed interest in implementing ABFT. For phase three, we planned to select 10 counselors from the phase two participants for further training and supervision based on (1) their (family) therapy training background, (2) their comfort in working with clients’ deep and vulnerable emotions, and (3) their service’s engagement to participate in the study.

Unfortunately, policy makers unexpectedly decided to reconfigure financial support for the project in response to the initial skepticism, concerns, and resistance that was strongly articulated by the counselors and administrations prior to the workshop. Nevertheless, two home-based services decided to implement ABFT. This created the opportunity to carry out an open trial study and to collect baseline and outcome data on all patients referred to ABFT services (Research goal 2). We aimed to explore implementation feasibility by investigating initial effectiveness of ABFT within home-based services of CWS. The target population was depressed adolescents and the primary outcomes were Children’s Depression Inventory (CDI; Kovacs, 2003), Youth Self-Report (YSR/11–18; Achenbach, 1991), and Child Behavior Checklist (CBCL/6–18; Achenbach, 1991).

## Method

### Research question 1: Does acceptability of EBP, i.e. ABFT, amongst CWS counselors increase after attending the introductory workshop?

*Participants*. The 73 workshop participants had work experience varying from zero to 37 years (*M* = 12.60, *SD* = 9.92). They had master’s degrees in psychology (32%), bachelor in special education (10%), bachelor in social work (28%), bachelor in psychology (2%), and bachelor in education (2%). Over half of the providers (64%) had advanced training in other modalities: cognitive behavioral therapy (15%), contextual therapy (13%), systems therapy (9%), psychodynamic therapy (1%), gestalt therapy (1%), structural family therapy (1%). The duration of these programs varied between zero and six years (*M* = 1.5 years, *SD =* 1.69 years).

*Procedure*. During the one-day introductory workshop, ABFT developers Drs. Guy S. Diamond and Suzanne A. Levy, together with ABFT staff member Dr. Torrey A. Creed, presented the CWS home-based counselors and administrators with lecture, clinical demonstrations and videotape excerpts of the clinical work. This workshop has been used around the world as the introductory format for training in ABFT. Additionally, for the last hour of the workshop, discussions between researchers and counselors about the ABFT model were organized. Specifically, a group of home-based administrators and a group of home-based counselors were asked to discuss with the ABFT team pitfalls and needs they experience in their current practices, and what they see as strengths and weaknesses of ABFT to effectively respond to those needs. Also, expected implementation barriers were discussed, and administrators’ and counselors’ concerns about the implementation process were addressed. Immediately before (Pre-measure) and after (Post-measure) the workshop, all participants were asked to complete an anonymous questionnaire assessing attitudes towards EBP and ABFT, fold the sheet and pass it to the front where the questionnaires were collected.

*Instruments*. A nine-item questionnaire was created to measure the attendees’ pre- and post-workshop attitudes towards EBPs in general (item 1 – 4) and ABFT specifically (item 5 – 9). The possible responses ranged on a Likert scale from one (total disagreement) to five (total agreement). These items are shown in Table 1. Four additional acceptability items were added to the Post-measure:

1. ‘ABFT would be a good fit for some of the families referred to the home-based services’.
2. ‘Now that I have seen what ABFT looks like in clinical practice, I would like further training in this model’. The possible responses ranged on a Likert scale from one (total disagreement) to five (total agreement).
3. ‘For what percentage of the families that you have already worked with, would ABFT have been a good treatment?’ Participants were asked to write down a percentage.
4. ‘Now that I have seen the ABFT presentation, my opinion about using ABFT in my work is more positive, not changed or more negative’. Participants were asked to select the answer which best represents their opinion.

**Table 1:** Pre- and Post-workshop measured attitudes per item for the total group.

Attitude items	*n*	Mean (*SD*) pre	Mean (*SD*) post	t (*df*)	*d*

1. I believe that interventional manuals could be used effectively by the families I work with	72	3.43 (*0.58*)	3.89 (*0.49*)	–6.00 (*71*)**	–0.86
2. I believe that ABFT fits within the work I am doing	73	3.70 (*0.76*)	3.86 (*0.69*)	–1.84 (*72*)	–0.22
3. I’m interested to learn how to do ABFT	73	4.22 (*0.73*)	4.29 (*0.68*)	–1.00 (*72*)	–0.10
4. I already know enough intervention techniques to guide most families effectively	72	2.46 (*0.80*)	2.32 (*0.80*)	1.46 (*71*)	0.18
5. Interventional manuals are too rigid for guiding families from our service	73	2.75 (*0.78*)	2.29 (*0.81*)	4.22 (*72*)**	0.58
6. Using a manual restricts my own style of working with families	73	2.73 (*0.90*)	2.26 (*0.88*)	3.68 (*72*)**	0.53
7. By using a manual I cannot deal as flexible with crisis	52	2.87 (*0.82*)	2.29 (*0.85*)	4.17 (*51*)**	0.69
8. Meaningful change cannot take place in 16 weeks	73	2.86 (*0.98*)	2.79 (*1.03*)	.52 (*72*)	0.07
9. ABFT focuses on the relation between the parent and one adolescent, and that is why it cannot be of additional value for all the other problems which the family has to deal with	72	2.33 (*0.96*)	2.19 (*1.05*)	.90 (*71*)	0.14

*Note*: * *p* < .006. ** *p* ≤ .001. .20 Small effect, .50 Medium effect, .80 Large effect (Cohen, 1988). To improve readability, we sorted the items based on the positively or negatively framed content.

### Research question 2: Do adolescent depressive symptoms decrease during home-based guidance by CWS services that implemented ABFT?

*Participants*. Over the course of five years, the Youth Protection Services (76.7%) and Juvenile Court (23.3%) referred 43 adolescents with depressive symptoms and/or dysfunctional family systems to the two ABFT clinics. The programs were set up in Ghent (48.8%) and Peer (51.2%). Of the 43 adolescents, 72.1% were female and 27.9% were male, and ranged from 11 to 17 years of age (*M* = 15.02, *SD* = 1.57). Participants came from two-parent families (18.6%), single-parent families (46.5%), co-parenting families (7%), blended families (14%), and adoptive families (2.3%). At baseline, 60.5% of adolescents endorsed internalizing problems above the subclinical range (23.3% above the clinical range).

*Procedure*. Adolescents and parent(s) were not randomly assigned to ABFT treatment, but rather invited by clinicians to engage in this treatment program. If they agreed, they provided informed consent to participate in the current study, which was approved by the Ethical Commission of the Catholic University of Leuven (KU Leuven). Expected duration of ABFT was six months. Actual duration (drop-outs excluded) lasted between five and nine months (*M* = 8.00, *SD* = 1.55). To measure treatment outcomes, we were given permission to access medical record data as collected by counselors to inform their daily clinical practice. Specifically, we were able to access screening questionnaires on depressive symptoms at baseline and post-treatment as filled out by parents and adolescents.

*Therapists*. ABFT was provided by eight community counselors, seven female and one male, ranging from 31 to 56 years of age (*M* = 41.75, *SD* = 9.94). They had master’s degrees in psychology (one counselor), master in criminology (one counselor), bachelor in social work (two counselors), and bachelor in special education (four counselors). Six counselors had advanced therapy training in other modalities: contextual therapy (four counselors), systems therapy (one counselor), and gestalt therapy (one counselor). The counselors’ work experience varied from five to 30 years (*M* = 17.25, *SD* = 9.72). We have no information on which therapist guided which case.

*ABFT training and supervision*. Counselors followed ABFT training consisting of a three-day introductory workshop, biweekly or monthly group supervision with the ABFT developers during the data collection phase, and a 3-day advanced workshop (six months later). We did not formally measure ABFT adherence.

*Instruments*. Internalizing problems and depressive symptoms were measured at baseline and post-treatment using the Children’s Depression Inventory (CDI), Youth self-report (YSR) and Child Behavior Checklist (CBCL).

*Children’s Depression Inventory*. The CDI (Kovacs, 2003; Dutch translation by Timbremont & Braet, 2002) is a 27-item self-rated scale to measure the severity of a child’s Depressive Symptoms. Participants selected the statement that best described how they felt about a symptom over the past two weeks (e.g. “I feel like crying every day/many days/sometimes.”). Each item is scored on a three-point scale, with higher scores reflecting more severity on that item. The CDI is a widely used measure, both for clinical and research purposes, of which reliability and validity have been extensively documented (Kovacs, 2003; Saylor, Spirito, & Bennett, 1984). In the current study, Cronbach’s αs of the CDI were 0.85 and 0.89 for pre and post-treatment measurement respectively.

*Youth self-report*. The YSR/11–18 (Achenbach, 1991) is a 112-item questionnaire used to assess a broad range of adolescent self-reported emotional and behavioral problems. Items are rated on a three-point Likert scale from zero to two. The measure consists of two Social Competence scales and eight Syndrome Scales, which can be grouped into two larger Internalizing/Externalizing scales. The current study focused on three scales: a) the Withdrawn/Depressed subscale (*α*_*pre*_ = 0.61; *α_post_* = 0.69), which denotes more detached behavior, b) the Anxious/Depressed subscale (*α*_*pre*_ = 0.83; *α_post_* = 0.85), which points to fearfulness and feelings of sadness, and c) the broadband Internalizing disorder scale (*α*_*pre*_ = 0.85; *α_post_* = 0.88).

*Child Behavior Checklist*. The CBCL/6–18 (Achenbach, 1991) respondent (usually parent or caregiver) identifies a broad range of child’s behavioral and/or emotional problems in a 118-item checklist. Reponses are recorded on a three-point Likert scale from zero to two. The current study included maternal reports^2^ and, similar to YSR, focused on three scales: a) the Withdrawn/Depressed Scale (*α*_*pre*_ = 0.65; *α_post_* = 0.77), b) Anxious/Depressed Scale (*α*_*pre*_ = 0.81; *α_post_* = 0.88), and c) the broadband Internalizing Disorder Scale (*α*_*pre*_ = 0.85; *α_post_* = 0.89). The CBCL and YSR are widely used measures, both for clinical and research purposes, of which reliability and validity have been extensively documented (e.g. Achenbach & Rescorla, 2001).

## Results

### Research question 1: Does acceptability of EBP, i.e. ABFT, amongst CWS counselors increase after attending the introductory workshop?

*Preliminary analyses*. On the pre assessment items, no data were missing. From the post-data .05% of the values on the questionnaire items was missing (51 items). Missing data were handled using pairwise deletion.

*Results*. Paired-samples *t*-tests were conducted to compare the nine Pre- and Post-measure items for the total sample. Cohen *d* effect sizes were calculated. Given the number of statistical tests we administered, Bonferroni correction for multiple testing was required. Effects are only to be considered as significant at *p* < .006. Table 1 shows administrators’ and counselors’ attitudes before and after participation in the introductory workshop for each acceptability item. Results showed a significant difference from pre- to post-measurement in favor of EBP for four of the acceptability items. Specifically, from pre- to post-measurement there was an increase in the belief that interventional manuals could be used effectively. Also, from pre to post, there was a decrease in the belief that interventional manuals restrict counselors’ personal style of guiding families, and are too rigid to be used with home-based families and to deal with crisis in a flexible way. For five of the items, no significant differences were found.

Additionally, we wanted to see if the workshop benefited attitudes of the counselors with initially the most negative attitudes (scores of three or less for positively framed items, and three or more for negatively framed items). Therefore, we repeated the same analyses for counselors with initially the most negative attitudes on each item. Results showed for all acceptability items a significant difference from pre- to post-measurement in favor of EBP (see Table 2).

**Table 2:** Pre- and Post-workshop measured attitudes per item for counselors with the most negative attitudes.

Attitude items	*n*	Mean (*SD*) pre	Mean (*SD*) post	t (*df*)	*d*

1. I believe that interventional manuals could be used effectively by the families I work with	40	2.95 (*0.22*)	3.78 (*0.48*)	–10.42 (*39*)**	–2.22
2. I believe that ABFT fits within the work I am doing	27	2.85 (*0.36*)	3.44 (*0.70*)	–3.65 (*26*)**	–1.06
3. I’m interested to learn how to do ABFT	11	2.91 (*0.30*)	3.45 (*0.52*)	–3.46 (*10*)*	–1.27
4. I already know enough intervention techniques to guide most families effectively	33	3.21 (*0.42*)	2.64 (*0.90*)	3.98 (*32*)**	0.81
5. Interventional manuals are too rigid for guiding families from our service	47	3.23 (*0.48*)	2.49 (*0.75*)	5.86 (*46*)**	1.18
6. Using a manual restricts my own style of working with families	46	3.30 (*0.51*)	2.43 (*0.83*)	6.49 (*45*)**	1.26
7. By using a manual I cannot deal as flexible with crisis	36	3.28 (*0.61*)	2.44 (*0.84*)	5.15 (*35*)**	1.14
8. Meaningful change cannot take place in 16 weeks	46	3.48 (*0.62*)	3.02 (*0.86*)	3.08 (*45*)*	0.61
9. ABFT focuses on the relation between the parent and one adolescent, and that is why it cannot be of additional value for all the other problems which the family has to deal with	26	3.42 (*0.58*)	2.38 (*1.02*)	4.48 (*25*)**	1.25

*Note*: * *p* < .006. ** *p* ≤ .001. .20 Small effect, .50 Medium effect, .80 Large effect (Cohen, 1988). To improve readability, we sorted the items based on the positively and negatively framed content.

Furthermore, the four additional acceptability questions were analyzed on a descriptive level. Scores on the four acceptability items showed that after attending the introductory workshop, on average, counselors and administrators:

1. believed ABFT would be a good fit for the families referred to the home-based service (*M* = 4.22, *SD* = 0.62, minimum = 3, maximum = 5).
2. thought ABFT would be a good treatment for almost half of the families they worked with (*M* = 46%, *SD* = 22%, minimum = 10%, maximum = 95%).
3. wanted further training in ABFT (*M* = 4.24, *SD* = 0.72, minimum = 2, maximum = 5).
4. had more positive opinions about ABFT after they had seen the ABFT presentation (not changed =13%, more negative = 1% [one participant]).

### Research question 2: Do adolescent depressive symptoms decrease during home-based guidance by CWS services that implemented ABFT?

*Preliminary analyses*. 23 of 43 adolescents completed YSR pre and post-treatment data, and 21 of those adolescents completed CDI pre and post-treatment data. Self-report post-treatment data were missing for 20 adolescents: four adolescents left the study because they were referred to a higher level of care, for two families the primary caregiver was no longer available to participate, six families dropped out because of lack of motivation for therapy by the adolescent and/or primary caregiver(s), and eight adolescents could not be contacted for post-assessment. Also, for those cases with completed self-report pre- and post-data, only 16 parents completed pre- and post-treatment questionnaires. When we compared baseline data on the main variables of the study for those who completed post-assessment and those who did not, we found no statistically significant differences on any of the primary outcome measures (0.20 ≤ *ps* ≤ 0.61).

To test for meaningful patterns in the missing data of the 23 adolescents with completed pre- and post-treatment data, we conducted the Little MCAR test (Chen & Little, 1988). This resulted in *χ*^2^ = 9997.47 (*df* = 12092; *p* = 1.00), which indicated that data were missing at random. Because data were only missing on the level of individual items, Mean Substitution was used to calculate scale scores. For the YSR, CBCL and CDI baseline data, missing values were replaced with the scale mean when 5% or less of the items were missing.

*Descriptive Statistics*. Correlations and mean level differences between mother- and adolescent-report (respectively CBCL and YSR) were calculated. Results of independent sample t-tests showed no significant mean level differences between mother- and adolescent-report for Internalizing problems, Withdrawn Depressed and Anxious Depressed at baseline and post-treatment (0.11 ≤ *p* ≤ 0.68) . Mother and child report were correlated at baseline for Internalizing Problems, *r*(18) = 0.70, *p* = 0.001, Withdrawn Depressed symptoms, *r*(18) = 0.59, *p* = 0.01, and Anxious Depressed symptoms, *r*(18) = 0.71, *p* = 0.001.

*Child report*. A paired-samples t-test was conducted to compare pre- and post-data of 23 adolescents. Results demonstrated significant decreases in Depressive Symptoms, Internalizing Problems, Withdrawn Depressed symptoms, and Anxious Depressed symptoms from baseline to post-treatment. As shown in Table 3, Cohen’s (1988) effect size values revealed medium to large effects.

**Table 3:** Child report: Pre- and Post-treatment measures.

Scale	*n*	Mean (*SD*) pre	Mean (*SD*) post	t (*df*)	*d*

Internalizing problems (YSR)	23	24.06 (*8.48*)	17.02 (*8.97*)	3.43 (*22*)**	.81
Withdrawn Depressed (YSR)	23	6.61 (*2.65*)	4.57 (*2.74*)	2.75 (*22*)*	.76
Anxious Depressed (YSR)	23	10.61 (*4.94*)	7.46 (*4.83*)	2.87 (*22*)**	.64
Depressive symptoms (CDI)	22	17.30 (*7.60*)	13.02 (*5.37*)	3.34 (*21*)**	.65

*Note*: * .01 < *p* ≤ .05. ** .001 < *p* ≤ .01. *** *p* ≤ .001. .20 Small effect, .50 Medium effect, .80 Large effect (Cohen, 1988).

*Mother report*. A paired-samples t-test was conducted to compare pre- and post-data of 16 mothers. Results revealed significant decreases in Internalizing Problems, Withdrawn Depressed symptoms, and Anxious Depressed symptoms from baseline to post-treatment. Cohen’s (1988) effect size values suggested medium effects (see Table 4).

**Table 4:** Parent report: Pre- and Post-treatment measures.

Scale	*n*	Mean (*SD*) pre	Mean (*SD*) post	t (*df*)	*d*

Internalizing problems (YSR)	16	19.54 (*10.26*)	13.26 (*8.97*)	2.63 (*15*)*	.65
Withdrawn Depressed (YSR)	16	5.94 (*3.00*)	3.73 (*2.92*)	3.38 (*15*)**	.75
Anxious Depressed (YSR)	16	9.27 (*5.30*)	5.36 (*5.10*)	4.62 (*15*)***	.75

*Note*: * .01 < *p* ≤ .05. ** .001 < *p* ≤ .01. *** *p* ≤ .001. .20 Small effect, .50 Medium effect, .80 Large effect (Cohen, 1988).

Finally, we tested whether there are differences between the two participating home-based services in terms of child- and mother-reported changes in symptoms from pre- to post-measurement. We created a variable indicating symptom decrease/increase for the main variables of the study (mean score symptoms pre-treatment minus mean score symptoms post-treatment). Positive scores reflect a decrease in symptoms, negative scores reflect an increase. One-way ANOVAs showed no significant differences between home-based services on changes in symptoms as reported by children (.39 ≤ *Fs* (1,21) ≤ 1.28, .27 ≤ *ps* ≤ .54) and mothers (.98 ≤ *Fs* (1,14) ≤ 1.96, .18 ≤ *ps* ≤ .34).

## Discussion

The current pilot study explored acceptability and feasibility of implementing ABFT in home-based services of the Flemish CWS to investigate the possibilities and challenges of EBP in CWS. The results showed that (1) several conceptions of EBP by CWS counselors were significantly more positive after attending the ABFT workshop, and that (2) adolescents and mothers reported significant decreases in adolescent depressive symptoms after receiving home-based treatment by CWS counselors that implemented ABFT.

### Research question 1: Does acceptability of EBP, i.e. ABFT, amongst CWS counselors increase after attending the introductory workshop?

The results suggested that the current sample’s CWS home-based counselors and administrators expressed similar skepticism and concerns as did therapists studied in previous research regarding implementing EBPs in community based settings (e.g. Addis, Wade, & Hatgis, 1999; Kazdin, 2008; Weisz & Gray, 2008; Weisz, Jensen-Doss, & Hawley, 2006). The current findings showed that after attending training in and active discussions about EBPs (i.e., ABFT), CWS counselors showed increased motivation to learn about these models and to use EBPs in their daily clinical practice. This is in line with Greenhalgh and colleagues’ (2004) observation that implementation success is conditional upon whether services and counselors feel that they are active participants from the start of the implementation process rather than passive recipients.

Although several perceptions regarding EBP by CWS counselors were significantly more positive after attending the ABFT workshop, no significant differences were found from pre- to post-measurement on five of the acceptability items. Nevertheless, additional analyses of the data of counselors who initially had the most negative attitudes for each item suggested that this could have been the result of ceiling effects. More specifically, the subgroup of counselors with initially the most negative attitudes reported significantly more positive attitudes towards EBPs and ABFT on all items after attending the workshop. Moreover, for this particular group, effect sizes were substantially larger than found for the total group. Interestingly, they also reported a significant decrease in confidence in their own knowledge about intervention techniques to guide families effectively after the workshop and discussions. The workshop may have increased their awareness that they could benefit from less known, additional intervention techniques to guide families effectively. The fact that our approach improved the attitudes of even the most critical individuals is important for future attempts to implement EBP because previous research showed that implementation success is conditional upon positive attitudes towards EBP (Addis, Wade, & Hatgis, 1999; Kazdin, 2008; Weisz & Gray, 2008; Weisz, Jensen-Doss, & Hawley, 2006). Moreover, the fact that both increases and decreases on distinct variables were noted for this group, precludes these effects to be interpreted in terms of methodological artifacts like regression to the mean.

Importantly, the lack of a comparison group in our design does not allow us to draw conclusions about a causal connection between the change in attitudes from pre- to post-measurement in favor of EBP and our implementation efforts. Additionally, one could argue that the positive changes in attitudes may reflect social desirability. However, given the attendants’ suspicion towards the treatment program about possible governmental hidden agendas, and given the fact that the attitude questionnaires were filled out anonymously, this seems less likely. On the contrary, if social desirability or the presence of other participants would have affected the post-measure, we would expect it to have resulted in increased negative attitudes given the general negative atmosphere of resistance. Furthermore, because we were not able to administer a follow-up questionnaire, we do not know how long these attitudes remained more positive. It would be interesting and important for further research to organize longer term follow-up measures of attitude change after the implementation of EBPs.

Although we cannot firmly conclude that changes in attitudes from pre- to post-measurement are due to attending the workshop nor will sustain over time, results of this exploratory study do suggest that providing training in EBP programs and organizing a platform for dialogue between developers of EBP programs and clinicians creates opportunities to overcome clinicians’ concerns regarding the application of EBP. This may suggest that the government’s decision to reconfigure financial support for the project in response to the initial skepticism and concerns prior to the workshop was premature.

### Research question 2: Do adolescent depressive symptoms decrease during home-based guidance by CWS services that implemented ABFT?

The results of the current study provided promising support that ABFT can be successfully used within home-based services of CWS to reduce depressive symptoms in adolescents as indicated both by adolescents and their mothers, showing medium to large effect sizes. Of course, without a control group, it is hard to say if usual care was just as effective. This will be followed up in future studies. Although promising, the current study’s self- and mother-reported decreases in depressive symptoms after ABFT treatment were substantially smaller than those found by Diamond and colleagues (2002) in a well-controlled efficacy study of ABFT for depressed adolescents (*d* = 1.21). Several factors may have contributed to this result. First, the complex reality and research conditions of community-based services are not comparable to the controlled environment in university-based intervention labs, the original development and testing context of efficacy trials. A treatment that is proven to be efficacious in a research setting, a controlled lab environment which allows treatment in optimal circumstances and with optimal control of confounding variables, does not automatically translate successfully to a “real-life” clinical setting (Addis & Krasnow, 2000; Hoagwood, Burns, Kiser, Ringeisen, & Schoenwald, 2001). The gap between EBPs and everyday clinical practice is a commonly voiced problem and it seems reasonable to assume that implementing ABFT in home-based services of the CWS has faced some similar challenges, and therefore, is more modest in its effects than previous efficacy trials. Therefore, future attempts to implement EBP should build on more thorough and systemic implementation strategies and implementation research.

Second, due to financial limitations, therapists of the current study were less tightly supervised and could not be fully supported and trained to become certified ABFT therapists. It would be helpful for future studies to have an objective assessment of the extent to which CWS counselors apply the ABFT treatment as intended. Novins and colleagues (2013) showed in their review examining implementation and dissemination efforts of EBPs that ongoing fidelity assessment, supervision, and support increase the likelihood that expected intervention effects are achieved. This implies that there could have been significant therapist effects on the treatment outcome. Unfortunately, we have no information about which therapist guided which case, and testing for therapist effects requires multi-level analyses that cannot be carried out reliably in the current study’s small sample.

Third, the current study suffered from a considerable amount of missing data Unfortunately, we had limited financial support and no research staff to carry out a rigorous data collection procedure. The small sample size limited the study’s statistical power and the mean substitution that was applied to missing items might have reduced variability, further reducing statistical power for the conducted tests.

In light of these limitations, the medium to large effect sizes suggest that ABFT may be an effective and promising approach to use within the context of home-based services of the CWS. However, a more rigorous research design and a larger sample are needed to draw stronger conclusions. Nevertheless, the fact that the current study’s results are in line with the RCT study in community clinics in Norway (Israel & Diamond, 2013) supports the relevance of our findings.

## Conclusion

The current study explored implementation possibilities of EBPs in CWS and showed preliminary but promising results concerning acceptability and feasibility. First, the current results suggest that active participation of counselors in training and discussions about EBPs may be an interesting strategy to overcome concerns and resistance towards implementation of EBP, especially for initial critics. Second, our results provided a first indication that EBPs, i.e. ABFT, in CWS services could be used for successful symptom reduction in clients. This was one of the first efforts in Flanders to implement an EBP in a CWS setting. During the implementation process, researchers, services, and government gained valuable insights about more optimal strategies to improve this process. The promising findings suggest that, in future, CWS and their clients could benefit from implementing more EBPs for a wider variety of problems. Additionally, it is important to use a “bottom-up” approach when implementing an EBP in a community setting. We conclude that adoption of EBPs in CWS is a promising and important path to further explore.

## Footnotes

Belgium is a federal state, consisting of three Communities based on the language: the Flemish, French and German- speaking Communities. Each Community has its own Government. Powers associated with the Communities are Culture, Education, Health Policy, Youth Protection, Social Welfare, etc.

Although fathers were also invited to complete the CBCL, they were not included given the limited amount of available data. Except for 5 cases, mothers were the only respondents.
